# Over-expression of HO-1 on mesenchymal stem cells promotes angiogenesis and improves myocardial function in infarcted myocardium

**DOI:** 10.1186/1423-0127-17-80

**Published:** 2010-10-07

**Authors:** Bin Zeng, Guosheng Lin, Xiaofeng Ren, Yan Zhang, Honglei Chen

**Affiliations:** 1 Department of Cardiology, Renmin Hospital of Wuhan University, Wuhan, Hubei, China; 2 College of Veterinary Medicine, Northeast Agricultural University, Harbin, Heilongjiang, China; 3 Department of Pathology, School of Basic Medical Science, Wuhan University, Wuhan, Hubei, China

## Abstract

Heme oxygenase-1 (HO-1) is a stress-inducible enzyme with diverse cytoprotective effects, and reported to have an important role in angiogenesis recently. Here we investigated whether HO-1 transduced by mesenchymal stem cells (MSCs) can induce angiogenic effects in infarcted myocardium. HO-1 was transfected into cultured MSCs using an adenoviral vector. 1 × 10^6 ^Ad-HO-1-transfected MSCs (HO-1-MSCs) or Ad-Null-transfected MSCs (Null-MSCs) or PBS was respectively injected into rat hearts intramyocardially at 1 h post-myocardial infarction. The results showed that HO-1-MSCs were able to induce stable expression of HO-1 *in vitro *and *in vivo*. The capillary density and expression of angiogenic growth factors, VEGF and FGF2 were significantly enhanced in HO-1-MSCs-treated hearts compared with Null-MSCs-treated and PBS-treated hearts. However, the angiogenic effects of HO-1 were abolished by treating the animals with HO inhibitor, zinc protoporphyrin. The myocardial apoptosis was marked reduced with significantly reduced fibrotic area in HO-1-MSCs-treated hearts; Furthermore, the cardiac function and remodeling were also significantly improved in HO-1-MSCs-treated hearts. Our current findings support the premise that HO-1 transduced by MSCs can induce angiogenic effects and improve heart function after acute myocardial infarction.

## Introduction

Recent pre-clinical and clinical studies have demonstrated that mesenchymal stem cells (MSCs) transplantation can attenuate ventricular remodeling and augment cardiac function when implanted into the infarcted myocardium. With an emerging interest to combine cell transplantation with gene therapy, MSCs are being assessed for their potential as carriers of exogenous therapeutic genes[1]. Several studies have showed that genetic modification of donor cells prior to transplantation may result in their enhanced survival, better engraftment and improved restoration in infarcted hearts. Genetic modification MSCs with antiapoptotic Bcl-2 gene enhanced the survival of engrafted MSCs in the heart after acute myocardial infarction, ameliorated LV remodeling and improved LV function[2]. Recent study shows that transplantation of MSCs transduced with Connexin43 gene into a rat MI model enhances MSCs survival, reduces infarct size, and improves contractile performance[3]. MSCs over-expressing Akt limit infarct size and improve ventricular function, and the functional improvement occurs in < 72 h[4]. However, improved survival of the cell graft may be less meaning if regional blood flow in the ischemic myocardium is not restored, especially expecting for long-term therapeutic effects.

HO-1 is a stress-inducible rate-limiting enzyme that catalyzes the breakdown of pro-oxidant heme into biliverdin, carbon monoxide (CO) and free iron. Biliverdin can be reduced to bilirubin by biliverdin reductase[5]. Several studies have shown that HO-1 is an anti-apoptotic and anti-oxidant enzyme, possessing cytoprotective activity under ischemic environment and increasing cell survival. Recently, studies have implicated a role for HO-1 in angiogenesis. Increasing expression of HO-1 can enhance proliferation and tube formation in human microvascular endothelial cells[6], and stromal cell-derived factor 1 promotes angiogenesis via a HO-1 dependent mechanism[7]. Furthermore, local HO-1 inhibition blocks angiogenesis[8]. Nevertheless, whether HO-1 transduced by MSCs has an effect on angiogenesis remains unclear. To test the hypothesis, we infected MSCs with recombinant adenovirus bearing human HO-1 (Adv-hHO-1) according to our previous protocols[9], and transplanted MSCs over-expressing HO-1 into acute myocardial infarction hearts. Our data indicate that over-expression of HO-1 in MSCs enhance angiogenesis and improves heart function in ischemic myocardium.

## Materials and methods

### Approval of animal experiments

The animal experiments were conformed to the Guide for the Care and Use of Laboratory Animals published by the US National Institute of Health (NIH published No.85-23, revised 1996).

### Preparation of recombinant adenovirus

A recombinant adenovirus containing human HO-1 (Adv-HO-1) was constructed as previously described [10]. Briefly, a full-length human HO-1 gene cDNA was cloned into the adenovirus shuttle plasmid vector pAd-CMV, which contains a cytomegalovirus promoter and a polyadenylation signal of bovine growth hormone. For construction of adenovirus containing green fluorescent protein (GFP), a shuttle vector containing human phosphoglycerate kinase gene promoter was used. The control virus lacking the hHO-1 gene (Adv-null) was separately prepared. Recombinant adenovirus was generated by homologous recombination and propagated in 293 cells. At stipulated time, the supernatant from 293 cells was collected and purified on cesium chloride (CsCl) gradient centrifugation and stored in 10 mmol/L Tris-HCl (pH 7.4), 1 mmol/L MgCl2, and 10% (vol/vol) glycerol at -70°C until used for experiments. Virus titers were determined by a plaque assay on 293 cell monolayers.

### Preparation of MSCs

MSCs were isolated from bone marrow of adult Sprague-Dawley male rats and expanded according to reported protocols [2,4]. Whole marrow cells were cultured at a density of 1 × 10^6 ^cells/cm^2 ^in α-minimum essential medium (α-MEM, Gibco, USA) with 10% fetal bovine serum (FBS, Invitrogen, USA) and 100 μg/ml penicillin-streptomycin (Sigma, USA). The nonadherent cells were removed by a medium change at 72 h and every four days thereafter. After two passages, homogeneous MSCs that devoid of hematopoietic cells were used. A total of 1 × 10^6 ^cells/ml MSCs were plated in plates for 24 h. The medium was then replaced with serum free α-MEM containing indicated multiplicities of infection (MOI) of Adv-HO-1 or Adv-null. After incubation for 2 h, an equal volume of α-MEM containing 20% FBS was added to the medium and cell culture was continued for another 48 hours. To observe the nuclei of MSCs in vitro, sterile 4',6'-diamidino-2' phenylindole (DAPI) (Sigma, USA) stock solution was added to culture medium at a final concentration of 50 μg/ml for 30 min. After labeling, cells were washed six times in D-Hanks solution to remove unbound DAPI and then the cells were observed using fluorescent microscopy.

### Cell implantation and trafficking of the MSCs *in vivo*

The male rats were anesthetized with sodium pentobarbital (40 mg/kg.i.p.), and mechanically ventilated. After the heart was exposed through a lateral thoracotomy, an 6-0 polypropylene thread was passed around the left coronary artery and the artery was occluded. Cyanosis and akinesia of the affected left ventricle were observed. The ECG was recorded to confirm the presence of infarction. One hour after myocardial infarction (MI), rats were randomly selected and approximately 1 × 10^6 ^HO-1MSCs or Null-MSCs in 0.1 ml of medium or equivalent volume of PBS alone was injected at four sites into the infarcted border zone using a 30-gauge needle (n = 12, each group). Some rats were given a daily intraperitoneal injection of the HO-1 inhibitor zinc-protoporphyrin (ZnPP, Porphyrin Products, Logan, UT, USA) at a concentration of 50 μmol/kg/day, starting two days before and continuing until 7 days after the HO-1-MSCs transplantation. Some rats were killed at 7 days after transplantation, and the treated hearts were harvested and cryopreserved in OCT media. Frozen tissue sections were used for histological examination of cell distribution.

### Western blot

MSCs were lysed in electrophoresis buffer (125 mmol/L Tris-HCl, pH 6.8, 12% glycerol, and 2% SDS), sonicated and boiled. Proteins (50 μg) were separated by sodium dodecyl sulfate polyacrylamide gel electrophoresis (SDS-PAGE), electrophoretically transferred to nitrocellulose membranes, and blocked with 1 × PBS containing Tween 20 (0.1%) and nonfat milk (5%) for 1 h. Then, the membranes were incubated with anti-HO-1 antibody (Santa Cruz, USA). Three weeks after transplantation, border regions of infarcted hearts from different groups were excised. Immunoblotting was performed using antibodies against VEGF or FGF2 (Santa Cruz, USA). Blots were developed by the ECL method (Pierce, USA), and relative protein levels were quantified by scanning densitometry and the relative gray value of protein = protein of interest/internal reference.

### RT-PCR

After 1 week of transplantation, the hearts was excised, and total RNA was extracted from the infarcted border zone using TRIzol reagent (Invitrogen, USA). The RT-PCR was performed as previously described [3].

### Immunohistochemistry

Three weeks after transplantation, myocardial specimens were embedded in OCT compound (Sigma), then quickly frozen in liquid nitrogen and stored at -80°C. Cryostat sections were cut into 5-μm. For immunostaining, sections were incubated with anti α-smooth muscle actin (abCAM, USA). The sections were then incubated with appropriate secondary antibody. Five fields per section were randomly selected and analyzed at a magnification of 200. The number of capillaries was assessed from photomicrographs by computerized image analysis.

### TUNEL Staining

To study the degree of cell apoptosis, TUNEL staining was performed using the In Situ Cell Death Detection Kit, POD (Roche, Germany) according to the manufacturer's instructions[11]. For each heart, the total number of TUNEL-positive myocyte nuclei in the infarcted zone was counted in ten sections. Individual nuclei were visualized at a magnification of 200, and the percentage of apoptotic nuclei (apoptotic nuclei/total nuclei) was calculated in 6 randomly chosen fields per slide and averaged for statistical analysis.

### Measurement of hemodynamics

4 weeks after injection, hemodynamic measurements were made. In brief, rats were anesthetized with pentobarbital sodium (60 mg/kg, i.p.). Catheter (model SPR-320, Millar, Inc.) filled with heparinized (10 U/ml) saline solution was placed in the right carotid artery and then advanced retrogradely into the LV. Hemodynamic parameters were recorded by a phyisiogical recorder (RJG-4122, Nihon Kohden, Japan).

### Assessment of Fibrosis

After 4 weeks of injection, the hearts were harvested, washed in PBS, and fixed in 10% formalin overnight at 4°C. Paraffin embedded tissues were cut into 5-μm sections and stained by Masson's Trichrome staining (Sigma) for collagen determination. Five fields per section were calculated and the collagen-delegated infarction percentage was analyzed by a blinded investigator. The calculation formula used for the infracted size is: % infarct size = infarct areas/total left ventricle area × 100%.

## Statistics

At least three independent experiments were carried out. Each data point was presented as mean ± SD. Statistical significance was evaluated using one-way ANOVA. A value of P < 0.05 was considered statistically significant.

## Results

### MSCs mediated HO-1 over-expression *in vitro *and *in vivo*

**Figure 1:**
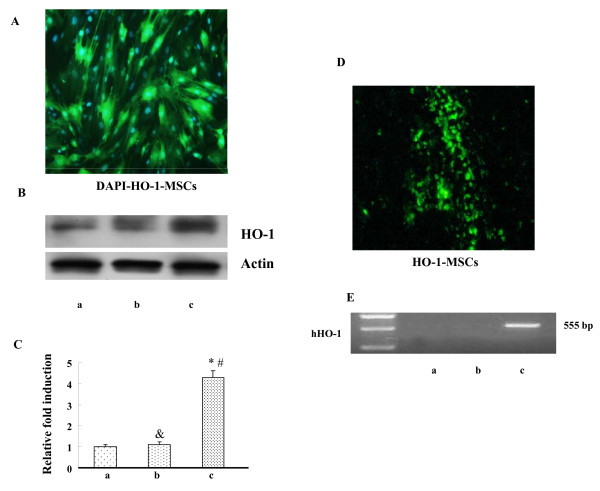
**HO-1 expression mediated by MSCs in Vitro and Vivo**. (A) HO-1 expression mediated by MSCs with GFP in Vitro (200×). (B) Western blot analysis of HO-1 protein in MSCs with actin used as an internal control. Lane a, MSCs control (untransfected); lane b, Null-MSCs; lane c, Adv-HO-1-MSCs. (C) Graph showing the relative fold induction of HO-1 protein levels in MSCs, n = 6. * P < 0.05 compared with MSCs control (untransfected); ^&^P > 0.05 compared with MSCs control (untransfected); ^# ^P < 0.05 compared with Null-MSCs. (D) Image from grafted HO-1-MSCs in the infarcted myocardium (200×). (E) RT-PCR detection mRNA in cardiac tissue. Lane a, MSCs control (untransfected); lane b, Null-MSCs; lane c, Adv-HO-1-MSCs.

MSCs isolated from rat bone marrow were infected with Adv-HO-1, and strong expression of GFP was observed by fluorescence analysis (Fig. 1A). The over-expression of HO-1 was confirmed by Western blotting (Fig. 1B). Levels of HO-1 in HO-1-MSCs were significantly higher than that in MSCs and Null-MSCs. At 7 days post-transplantation, the HO-1-MSCs were embedded into the host myocardium (Fig. 1C). The expression of HO-1 in hearts was confirmed by relative quantification of hHO-1 mRNA (Fig. 1D). The hHO-1 mRNA was detected in the cardiac sample extracted from cardiac tissue of HO-1-MSCs group rather than in the Null-MSCs and PBS group.

### Effects of HO-1-MSCs transplantation on angiogenesis

**Figure 2:**
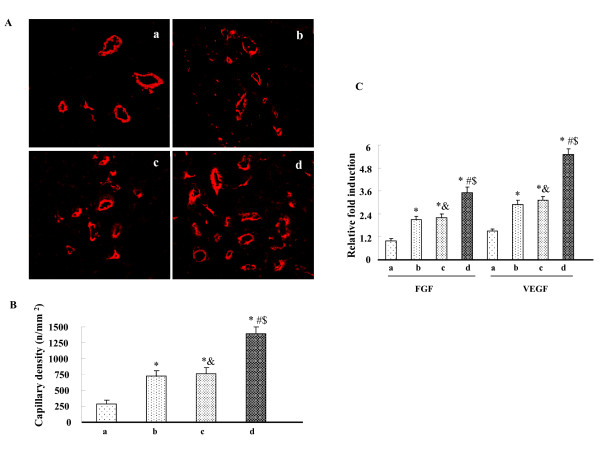
**Effects of HO-1-MSCs transplantation on neovascularization and angiogenic growth factors**. (A) Representative microvessel in the border of infarcted myocardium 3 weeks after transplantation (200×). (B) Values are means ± SD of data from 6 separate experiments, * P < 0.05 compared with the hearts treated with PBS. ^# ^P < 0.05 compared with the hearts treated with Null-MSCs. ^&^P > 0.05 compared with the hearts treated with Null-MSCs. ^$ ^P < 0.05 compared with the hearts treated with HO-1-MSCs and HO inhibitor. Lane a, hearts treated with PBS; Lane b, hearts treated with Null-MSCs; Lane c, hearts treated with HO-1-MSCs and HO inhibitor; Lane d, hearts treated with HO-1-MSCs. (C) Blots regarding the expression of FGF2, VEGF and actin were developed by the ECL method and relative protein levels were quantified by scanning densitometry and the relative gray value of protein = protein of interest/internal reference. Values are means ± SD of data from 6 separate experiments, * P < 0.05 compared with the hearts treated with PBS. ^# ^P < 0.05 compared with the hearts treated with Null-MSCs. ^&^P > 0.05 compared with the hearts treated with Null-MSCs. ^$ ^P < 0.05 compared with the hearts treated with HO-1-MSCs and HO inhibitor. Lane a, hearts treated with PBS; Lane b, hearts treated with Null-MSCs; Lane c, hearts treated with HO-1-MSCs and HO inhibitor; Lane d, hearts treated with HO-1-MSCs.

Immunofluorescent staining for α-smooth muscle actin and quantification of capillary density revealed that the capillary density was significantly enhanced by HO-1-MSCs transplantation compared with Null-MSCs and PBS transplantation; and the capillary density was also significantly enhanced by Null-MSCs transplantation compared with by PBS transplantation (Fig 2A, B). To determine whether expression of HO-1 mediated by MSCs results in angiogenesis and to minimize the impacts on angiogenesis induced by MSCs in this study, we investigated the effect of an HO inhibitor, ZnPP, on the HO-1-MSCs group. ZnPP treatment abolished the increase in capillary density. There was not significant difference between Null-MSCs group and ZnPP treated HO-1-MSCs group (Fig 2A, B). Similarly, the expressions of angiogenic factors VEGF and FGF2 were significantly higher in HO-1-MSCs group compared with Null-MSCs group and ZnPP treated HO-1-MSCs group; The expression of VEGF and FGF2 did not differ between Null-MSCs group and ZnPP treated HO-1-MSCs group (Fig. 2C).

### Effects of HO-1-MSCs transplantation on myocyte apoptosis

**Figure 3:**
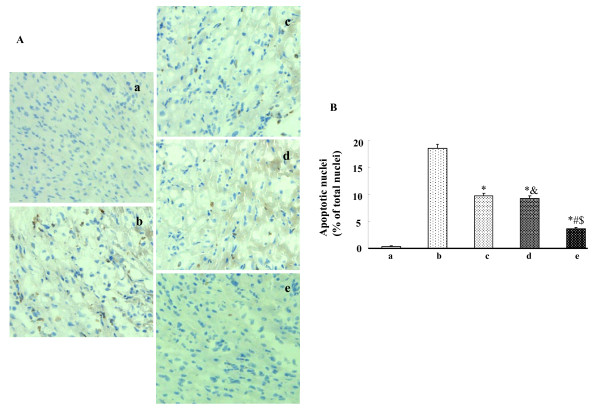
**Effects of HO-1-MSCs transplantation on apoptosis**. (A) TUNEL-positive cells in the border zone of infracted myocardium 3 weeks after transplantation (100×). (B) Values are means ± SD of data from 6 separate experiments, * P < 0.05 compared with the hearts treated with PBS. ^# ^P < 0.05 compared with the hearts treated with Null-MSCs. ^&^P > 0.05 compared with the hearts treated with Null-MSCs. ^$ ^P < 0.05 compared with the hearts treated with HO-1-MSCs and HO inhibitor. Lane a, normal control; Lane b, hearts treated with PBS; Lane c, hearts treated with Null-MSCs; Lane d, hearts treated with HO-1-MSCs and HO inhibitor; Lane e, hearts treated with HO-1-MSCs.

The degree of myocyte apoptosis as assessed by TNUEL was significantly less in the HO-1-MSCs group than other groups, and there was no significant difference between Null-MSCs group and ZnPP treated HO-1-MSCs group. TUNEL positive nuclei were also less in Null-MSCs group and ZnPP treated HO-1-MSCs group than that in PBS group (Fig. 3A, B).

### Effects of HO-1-MSCs transplantation on ventricular function and fibrosis

**Figure 4:**
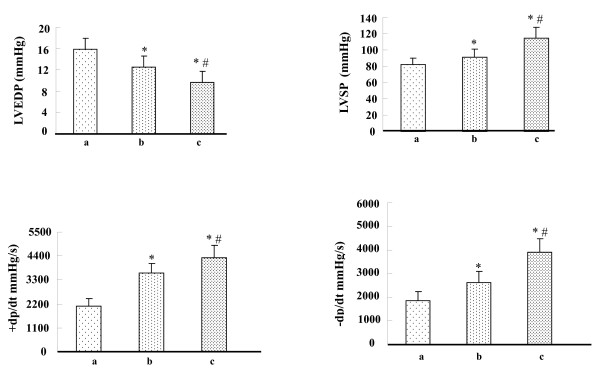
**Effects of HO-1-MSCs transplantation on ventricular function**. (A) Hemodynamic assessment of cardiac function at 4 weeks after transplantation. LVSP: left ventricle systolic pressure; LVEDP: left ventricle end-diastolic pressure; + dP/dtmax and -dP/dtmax: rate of rise and fall of ventricular pressure, respectively. means ± SD of data from 6 separate experiments, *P < 0.05 compared with the hearts treated with PBS, ^#^P < 0.05 compared with the hearts treated with Null-MSCs. Lane a, hearts treated with PBS; Lane b, hearts treated with Null-MSCs; Lane c, hearts treated with HO-1-MSCs.

**Figure 5:**
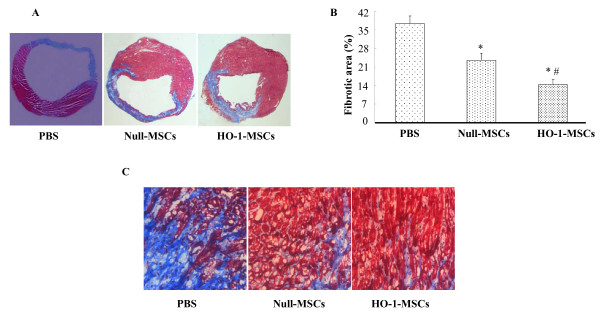
**Effects of HO-1-MSCs transplantation on ventricular remodeling**. (A) The transmural slices of the left ventricle were stained with Masson trichrome (1.25×). (B) % fibrotic area in heart with infarction was measured. Values are means ± SD of data from 6 separate experiments, *P < 0.05 compared with the hearts treated with PBS, ^#^P < 0.05 compared with the hearts treated with Null-MSCs. (C) The border zone of the infarct area (100×).

Hemodynamic parameters were measured 4 weeks after transplantation. LV function in HO-1-MSCs and Null-MSCs group was improved significantly compared with that in PBS group, and there was significant difference between HO-1-MSCs and Null-MSCs group (Fig. 4). The typical left ventricle wall sections after Masson-Trichome staining were shown on Fig. 5A, C. The percentage of fibrosis in the HO-1-MSCs and Null-MSCs group was significantly reduced compared with PBS group, which was the lowest in HO-1-MSCs group (Fig. 5B).

## Discussion

Under most circumstance, the treatment of MI by using MSCs showed poor survival of transplanted cells. In addition to the quick loss of cells within 24 h of transplantation caused by cell leakage into the extra myocardial space, or being flushed out in the coronary vein, the molecular mechanism for cell death in ischemic myocardium may include ischemia, ischemic/reperfusion, and more importantly the host inflammatory response mediators and proapoptotic factors in the ischemic myocardium. It has been showed that inflammatory process after MI peaks at 1 week[12], and apoptosis is a major factor causing donor cell death[13]. Many studies point to the anti-apoptotic and anti-inflammatory effects. It is clear that angiogenesis cannot only improve the survival of transplanted cells, but also reduce myocardial apoptosis and restores the heart function.

MSCs were reported to have the potential to release several kinds of cytokines, which induce angiogenesis[4,9]. However, the number of cells at 3 weeks after transplantation decreased significantly, and almost all transplantation cells seemed to be lost at 6 weeks[14]. Limited MSCs cannot achieve maximum functional benefits of angiogenesis. HO-1 has been recognized to be involved in diverse cytoprotective effects, due to its multiple catalytic byproducts. HO-1 was administered to improve the survival environment of MSCs and to achieve maximum functional benefits of MSCs[15]. Recent studies showed that over-expression of the HO-1 gene in endothelial cell caused a significant increase in angiogenesis[16]. Adenovirus-mediated HO-1 gene transfer into the ischemic hindlimb facilitated a significant recovery of blood flow in the hindlimb, and this effect was, at least in part, due to an increase in the capillary density, thus, to angiogenic effects of HO-1[17]. In our study, capillary density and the expression of angiogenic growth factors, including vascular endothelial growth factor (VEGF) and fibroblast growth factor 2 (FGF2), in the border area of the infarct in HO-1-MSCs group was significantly higher than that in Null-MSCs group and ZnPP treated HO-1-MSCs group. However, capillary density and the expression of VEGF and FGF2 did not show significant difference between Null-MSCs and ZnPP treated HO-1-MSCs group, indicating the role of HO-1 in the induction of angiogenesis. We confirmed that HO-1 transduced by MSCs also have positive effects on angiogenesis. It has been reported that nitric oxide (NO) may modulate angiogenesis by upregulating VEGF in vascular cells, and NO inhibitors can reduce the angiogenic potential of endothelial cells[18]. CO may also be involved in the expression of VEGF[19]. Another contributor to enhance angiogenesis may be the increasing expression of angiogenic growth factors in the ischemic myocardium. VEGF is a strong therapeutic reagent by inducing angiogenesis in ischemic myocardium[20], and VEGF can mediate the ischemia-induced mobilization of bone marrow stem cells[21]. In addition, FGF2 also have the potential to promote angiogenesis, and regulate proliferation, migration, differentiation of vascular cells[22,23]. Lin'study showed that HO-1 gene transfer post MI provides protection at least in part by promoting angiogenesis through inducing angiogenic growth factors[24].

Angiogenesis contributes to the regional blood flow in the ischemic myocardium. Cardiomyocytes death plays an important role in the development of remodeling; ventricular remodeling with chamber dilatation and wall thinning are important features of post-infarction cardiac function[25,26]. Studies have shown that late reperfusion after infarction results in enhanced cardiac function and remodeling. The improved blood supply may result in salvaging of cardiomyocytes that would otherwise be lost or no-functional due to ischemia. In addition, VEGF, may provide myocardial protection, blocking the programmed cell death response that is know to contribute significantly to the development of ischemic heart failure[27,28]. In the current study, significant decrease of apoptotic cells in HO-1-MSCs group was observed as compared with that of control groups, and the enlargement of LV dilatation and fibrosis were significantly decreased in HO-1-MSCs group with smaller chambers and thicker LV anterior walls. Echocardiographic results further confirmed our hypothesis that HO-1 modified MSCs significantly improve LV function.

In conclusion, HO-1 transduced by MSCs can induce angiogenic effects and improve heart function after acute myocardial infarction

## Competing interests

The authors declare that they have no competing interests.

## Authors' contributions

BZ designed, carried out the main experiment and drafted the manuscript. GS-L helped to design the experiment and drafted the manuscript. XF-R helped to finish the statistical analysis and improve the manuscript. YZ participated in RT-PCR and Western blot analysis. HL-Ch helped to finish histological experiments. All authors read and approved the final manuscript.
